# Pd@Pt Nanodendrites as Peroxidase Nanomimics for Enhanced Colorimetric ELISA of Cytokines with Femtomolar Sensitivity

**DOI:** 10.3390/chemosensors10090359

**Published:** 2022-09-08

**Authors:** Zhuangqiang Gao, Chuanyu Wang, Jiacheng He, Pengyu Chen

**Affiliations:** Materials Research and Education Center, Materials Engineering, Department of Mechanical Engineering, Auburn University, Auburn, Alabama 36849, United States

**Keywords:** cytokine, interleukin-6, enzyme-linked immunosorbent assay, ultrasensitive colorimetric detection, Pd@Pt core@shell nanodendrites, peroxidase nanomimics

## Abstract

Colorimetric enzyme-linked immunosorbent assay (ELISA) has been widely applied as the gold-standard method for cytokine detection over decades. However, it has become a critical challenge to further improve the detection sensitivity of ELISA as limited by the catalytic activity of enzymes. Herein, we report an enhanced colorimetric ELISA for ultrasensitive detection of interleukin-6 (IL-6, as a model cytokine for demonstration) using Pd@Pt core@shell nanodendrites (Pd@Pt NDs) as peroxidase nanomimics (named “Pd@Pt ND ELISA”), pushing the sensitivity up to femtomolar level. Specifically, the Pd@Pt NDs are rationally engineered by depositing Pt atoms on Pd nanocubes (NCs) to generate rough dendrite-like Pt skins on the Pd surfaces via Volmer-Weber growth mode. They can be produced on a large scale with highly uniform size, shape, composition, and structure. They exhibit significantly enhanced peroxidase-like catalytic activity with catalytic constants (${\text{K}}_{\text{cat}}$) more than 2000-fold higher than those of horseradish peroxidase (HRP, an enzyme commonly used in ELISA). Using Pd@Pt NDs as the signal labels, the Pd@Pt ND ELISA presents strong colorimetric signals for the quantitative determination of IL-6 with a wide dynamic range of 0.05–100 pg mL-1 and an ultralow detection limit of 0.044 pg mL-1 (1.7 fM). This detection limit is 21-fold lower than that of conventional HRP-based ELISA. The reproducibility and specificity of the Pd@Pt ND ELISA are excellent. More significantly, the Pd@Pt ND ELISA was validated for analyzing IL-6 in human serum samples with high accuracy and reliability through recovery tests. Our results demonstrate that the colorimetric Pd@Pt ND ELISA is a promising biosensing tool for ultrasensitive determination of cytokines and thus is expected to be applied in a variety of clinical diagnoses and fundamental biomedical studies.

## 1. Introduction

Cytokines are a class of bioactive proteins with molecular weights of 6–70 kDa, secreted by immune cells (*e.g.*, T lymphocytes, B lymphocytes, macrophages, monocytes, natural killer cells, and mast cells), epidermal cells, endothelial cells, fibroblasts, etc. [1–4]. As cell signaling molecules, they participate in a diverse and broad spectrum of biological activities, such as cell-to-cell communication, modulation of immune responses, as well as regulation of cell maturation, growth, development, and differentiation [5–8]. As disease biomarkers, they are strongly associated with the immune status of hosts in many diseases, such as infection, inflammation, trauma, lupus, sepsis, and cancer [4,9–12]. For example, interleukin-6 (IL-6) is a pleiotropic cytokine that plays an important role in host defense by modulating immune and inflammatory responses. It activates various immune-related biological effects, *e.g.*, the induction of B lymphocyte differentiation to produce immunoglobulin, the promotion of T lymphocyte proliferation and growth, and the enhancement of blood cell differentiation, through cytokine network and cell-to-cell communication, thereby motivating the immune system for host defense [13–15]. It also induces the production of C-reactive protein and procalcitonin and is directly related to the inflammatory status in patients, thus serving as a key biomarker for early diagnosis of inflammatory diseases, *e.g.*, the prediction of occurrence of sepsis and cytokine storm syndrome [10,13,16–19]. Therefore, obtaining information about qualitative and quantitative nature of cytokine expression and release is crucial for understanding immune-related physiological and pathological processes and facilitating early diagnosis and treatment of inflammatory diseases.

Colorimetric enzyme-linked immunosorbent assay (ELISA) has been broadly recognized as the gold-standard analytical method for cytokine detection due to its competitive advantages in simplicity, practicality, low cost, and easy operation [4,11,20,21]. However, the major drawback of conventional colorimetric ELISA is the relatively low sensitivity with the detection limits in the range of 0.7–1,437 pg/mL (*i.e.*, 26.9–145,152 fM, see Table S2) [22,23], making it insufficient for monitoring cytokines at ultra-low levels (*e.g.*, fM level). It should be emphasized that in many cases, the levels of expressed/released cytokines in biological samples are typically below the detection limit of conventional colorimetric ELISA, and the improvement of its sensitivity has become essential to acquire more valuable information about the immune status with cytokines at ultra-low levels [3,4,11,24,25]. Because the detection signal of colorimetric ELISA mainly originates from the catalysis of enzymes [*e.g.*, horseradish peroxidase (HRP)] toward substrates to produce colored products, the detection sensitivity is essentially confined by the inherent catalytic efficiency of enzymes. Specifically, enzymes with higher catalytic efficiency can produce more colored products, thus generating stronger colorimetric signals for more sensitive detection. As a result, a lower concentration of cytokines can be detected. Hence, there is an urgent need to explore new catalysts with higher catalytic efficiency to further improve the sensitivity of colorimetric ELISA for ultrasensitive cytokine detection.

Recent studies on peroxidase mimics made of inorganic nanostructures (*i.e.*, peroxidase nanomimics) have unveiled their potential in advanced bioassay systems [26–29]. Through rationally controlling the size, shape, composition, and structure, the catalytic properties of peroxidase nanomimics can be precisely tailored to meet the requirements of advanced bioassays. The first peroxidase nanomimic was reported by the Yan group with the discovery of peroxidase-like catalytic activity of ${\text{Fe}}_{3}{\text{O}}_{4}$ nanoparticles in 2007 [30]. Since then, a variety of inorganic nanostructures have been actively reported to possess peroxidase-like catalytic properties [26–29]. Notable examples include metal oxide nanomaterials (*e.g.*, ${\text{Co}}_{3}{\text{O}}_{4}$ nanoparticles, ${\text{MnO}}_{2}$ nanowires, and ${\text{V}}_{2}{\text{O}}_{5}$ nanowires) [31–33], metal sulfide nanomaterials (*e.g.*, ${\text{MoS}}_{2}$ and ${\text{WS}}_{2}$ nanosheets) [34,35], carbon nanomaterials (graphene oxide and carbon nanotubes) [35,36], and noble metal nanomaterials (*e.g.*, Au, Ag, Pd, Pt, Ir, and Ru nanoparticles) [37–42]. Compared with natural peroxidases, these peroxidase nanomimics have some demerits including increased toxicity, decreased accessibility, and more complicated considerations regarding waste disposal procedures, but they show interesting merits including high catalytic activity, excellent stability, facile synthesis, and easy storage, making them appealing candidates for peroxidases in bioassays [26–29]. Among these nanostructures, Pt nanodendrites (NDs) have recently emerged as an innovative peroxidase nanomimic for ultrasensitive colorimetric immunoassays because of several more important advantages [43–49]. First, the nanoparticles made of Pt element show a much higher peroxidase-like catalytic activity than those of other elements. Second, the rough dendrite-like surface structure offers more active sites for the catalytic reaction. Third, the nanoparticles can be easily functionalized with various functional molecules through thiol-Pt chemistry. Thus, the development of Pt ND peroxidase nanomimics as an alternative to enzyme labels has become a viable approach to greatly improve the sensitivity of colorimetric ELISA for cytokine detection. However, it has been challenging to large-scale production of Pt NDs while finely controlling the uniformity of their size and shape, which seriously hinders their widespread implementation in sensitive cytokine immunoassays.

**Scheme 1.**
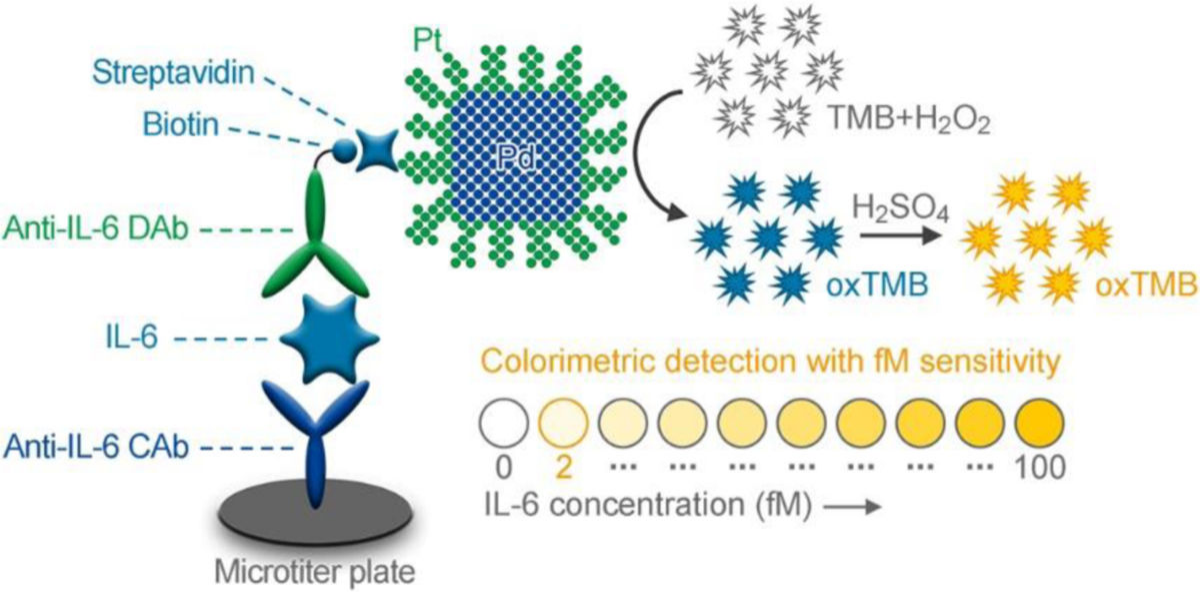
Schematic illustration of the enhanced colorimetric ELISA for ultrasensitive detection of IL-6 using Pd@Pt ND peroxidase nanomimics as the signal labels.

In this work, we developed an enhanced colorimetric ELISA for ultrasensitive detection of IL-6 (as a model cytokine) by employing Pd@Pt core@shell nanodendrites (Pd@Pt NDs) as the peroxidase nanomimics (named “Pd@Pt ND ELISA”, Scheme 1). The Pd@Pt NDs were synthesized by coating Pd nanocubes (Pd NCs) with a layer of rough dendrite-like Pt skins on the surfaces. Different from the previously reported dendrite-like Pt nanoparticles, the Pd@Pt NDs can be mass-produced with high uniformity in terms of both size and shape, showing highly efficient peroxidase-like activity with catalytic constants ($K_{\text{cat}}$) up to 7–9 × 10^6^ s^−1^, 2000-fold higher than those of HRP. The immunoassay was carried out in anti-IL-6 capture-antibody- (CAb-) immobilized microplate plates by coupling with biotin-conjugated anti-IL-6 detection antibody (biotin-anti-IL-6 DAb) as the recognition element and Pd@Pt ND-labeled streptavidin (Pd@Pt ND-SA) as the signal probe. In this way, the sandwich-type immunocomplex (*i.e.*, anti-IL-6 CAb/IL-6/biotin-anti-IL-6 DAb/Pd@Pt ND-SA) was formed on the microplate plates. Upon addition of substrates [*i.e.*, 3,3’,5,5’-tetramethylbenzidine (TMB) and hydrogen peroxide (${\text{H}}_{2}{\text{O}}_{2}$)], the labeled Pd@Pt NDs catalyzed the oxidation of TMB by ${\text{H}}_{2}{\text{O}}_{2}$ to generate an intense colorimetric signal for ultrasensitive detection of IL-6. Using conventional HRP-based ELISA as a benchmark, we demonstrate that the sensitivity of our Pd@Pt ND ELISA could be enhanced by one order of magnitude (21 times).

## 2. Materials and Methods

### 2.1. Preparation of 18 nm Pd NCs.

The Pd NCs with an edge length of 18 nm were prepared using a simple one-pot synthesis protocol with some modifications [50]. Initially, 4.0 mL of 52.5 mg/mL poly(vinylpyrrolidone) (PVP, ${\mathrm{Na}}_{\text{W}}$ ≈ 55,000) aqueous solution, 4.0 mL of 30 mg/mL L-ascorbic acid (AA) aqueous solution, and 8.0 mL of 150 mg/mL KBr aqueous solution were hosted in a 40-mL glass vial and preheated at 80 °C under magnetic stirring for 20 min. Then, 6.0 mL of 19 mg/mL ${\text{Na}}_{2}{\text{PdCl}}_{4}$ aqueous solution was added to the vial. The mixed solution was incubated at 80 °C for 3 h. After cool-down, the 18 nm Pd NCs as products were collected *via* centrifugation, and re-dispersed in 20 mL of DI water in a 40-mL glass vial for further use (19.4 mM of Pd atomic molarity, 48.8 nM of particle concentration).

### 2.2. Synthesis of Pd@Pt NDs.

The Pd@Pt NDs were synthesized using a facile seed-mediated growth procedure with the as-prepared 18 nm Pd NCs as the seeds. In a standard synthesis, 300 μL of the 18 nm Pd NCs suspension was added to 5.0 mL of 0.02% PVP aqueous solution in a 20-mL glass vial. Subsequently, 100 μL of 50 mM ${\text{Na}}_{2}{\text{PtCl}}_{6}$ aqueous solution and 600 μL of 100 mM AA aqueous solution were added to the vial in sequence, followed by vigorous shaking. The mixed solution was allowed to react at 80 °C in an oven for 3 h and then cooled down to room temperature. After being washed once with DI water *via* centrifugation, the Pd@Pt NDs as products were stored in 2.4 mL of DI water in a 20-mL glass vial for future use (6.10 nM in particle concentration). The molar ratio of Pt to Pd for this Pd@Pt ND sample was 0.860.

To synthesize Pd@Pt NDs with different molar ratios (*x*) of Pt to Pd (*i.e.*, ${\text{Pd@Pt}}_{x}$ NDs, *x* = 0–3.44), similar procedures as above were used except for adding varied volumes of 50 mM ${\text{Na}}_{2}{\text{PtCl}}_{6}$ aqueous solution in the range of 0–400 μL.

For the 60-fold scaled-up synthesis, the synthesis procedure remained the same except that the volumes of all reagents were increased 60-fold and the 20-mL glass vial was changed to a 500-mL round-bottom flask. This scaled-up synthesis could result in the production of 144 mL of 6.10 nM Pd@Pt ND aqueous suspension, which contains 37.1 mg of Pd and 58.5 mg of Pt (95.6 mg in total).

### 2.3. Apparent steady-state kinetic assays.

The steady-state kinetic assays were performed according to our recent reports with minor modifications [51,52]. In brief, a series of 1-mL substrate solutions with different concentrations of TMB and ${\text{H}}_{2}{\text{O}}_{2}$ were prepared in citrate-phosphate buffer (pH 4.0) in cuvettes with a path length (*l*) of 1.0 cm at room temperature. For kinetic assays toward TMB, the ${\text{H}}_{2}{\text{O}}_{2}$ concentration was fixed at 7.0 M, and the TMB concentration was altered in the range of 0.02–0.8 mM. For kinetic assays toward ${\text{H}}_{2}{\text{O}}_{2}$, the TMB concentration was fixed at 0.8 mM, and the ${\text{H}}_{2}{\text{O}}_{2}$ concentration was altered in the range of 0.2–7.0 M. Subsequently, 1 μL of Pd@Pt NDs (0.0244 nM) was added to each of the substrate solutions and mixed thoroughly. Right after mixing the substrate solution with Pd@Pt NDs, the absorbance (at 652 nm) of each reaction solution was measured as a function of time with intervals of 5 s for 5 min using a UV-vis spectrophotometer. The obtained “absorbance vs time” curve was the kinetic curve of each reaction. The slope at the initial point (${\text{Slope}}_{\text{Initial}}$) of the kinetic curve was calculated using OriginPro 2021b software, and thus the initial rate (*ν*) of each reaction could be calculated by the equation: $v={\text{Slope}}_{\text{Initial}}/\left(\varepsilon_{0\mathrm{xTMB}-652\;\text{nm}}\times l\right)$, where $\varepsilon_{\text{oxTMB-652nm}}$ is the molar absorption coefficient of oxidized TMB (oxTMB) at 652 nm ($\varepsilon_{0\text{xTMB}-652\;\text{nm}}=3.9\times 10^{4}{\text{M}}^{-1}{\text{cm}}^{-1}$). Finally, the apparent steady-state kinetic parameters (including $V_{\max}$, $K_{m}$, and $K_{\text{cat}}$) could be obtained from the double-reciprocal plots of v *versus* substrate concentrations, the Lineweaver-Burk equation: $1/v=K_{m}/V_{\max}\;1/[S]+1/V_{\max}$, and the catalytic constant equation: $K_{\text{cat}}=V_{\max}/[E]$, where $V_{\max}$, [*S*], $K_{\text{m}}$, $K_{\text{cat}}$, and [*E*] represent the maximal reaction rate, the concentration of substrate, the Michaelis constant, the catalytic constant, and the concentration of catalyst, respectively.

### 2.4. Preparation of streptavidin-conjugated Pd@Pt NDs (Pd@Pt ND-SA conjugates).

The Pd@Pt ND-SA conjugates were prepared through chemical modifications based on our recently published procedures with some modifications [51,53]. Briefly, 200 μL of the Pd@Pt NDs (6.10 nM) was mixed with 200 μL of 20 mg mL^−1^ HS-PEG-COOH aqueous solution. The mixture was incubated at room temperature for 3 h under gentle shaking, followed by centrifugation at 13,000 rpm for 20 min. The obtained nanoparticles were washed twice with DI water and then redispersed in 400 μL of 10 mM phosphate-buffered saline (PBS, pH 7.4). After that, 100 μL of an aqueous solution containing 25 mM EDC and 50 mM NHS was added to the nanoparticle suspension. After incubation at room temperature for 20 min, the nanoparticles were collected *via* centrifugation, washed twice with DI water, and redispersed in 500 μL of PBS (pH 7.4). Subsequently, 10 μL of 1 mg mL^−1^ streptavidin (SA) was added to the nanoparticle suspension, and the resulting mixture was incubated at 4 °C in a fridge overnight with gentle shaking. Then, 200 μL of 10% BSA in PBS (pH 7.4) was added to the reaction suspension. After incubation at room temperature for 60 min, the products (*i.e*., Pd@Pt ND-SA conjugates) were washed with PBST [PBS (pH 7.4) containing 0.05% Tween 20] three times *via* centrifugation, redispersed in 100 μL of PBST containing 1% BSA and 0.02% ${\text{NaN}}_{3}$, and finally stored at 4 °C in a fridge for future use (12.2 nM).

### 2.5. Colorimetric Pd@Pt ND ELISA of IL-6.

Before detection, the 96-well microtiter plates were coated with 50 μL of 2.5 μg mL^−1^ anti-IL-6 CAb in 10 mM carbonate-bicarbonate buffer (pH 9.6) at 4 °C overnight. After washing with washing buffer (PBST) five times, the plates were blocked by 350 μL of block-fix buffer (PBST containing 1% BSA and 15% sucrose) at room temperature for 3 h. Then, the block-fix buffer was aspirated, and the plates were dried at room temperature. After being sealed with desiccant, the plates were stored at 4 °C in a fridge for further use. In a standard assay procedure, 100 μL of IL-6 standards with different concentrations in dilution buffer (PBST containing 1% BSA) and 50 μL of 0.2 μg mL^−1^ biotin-anti-IL-6 DAb in dilution buffer were sequentially added to each well of the plates. After incubation at room temperature for 2 h under shaking, the plates were washed five times with washing buffer, followed by the addition of 100 μL of Pd@Pt ND-SA conjugates (0.122 nM) in dilution buffer into each well. After incubation at room temperature for 30 min under shaking, the plates were washed again. Afterward, 100 μL of substrate solution [0.8 mM TMB and 7.0 M ${\text{H}}_{2}{\text{O}}_{2}$ in citrate-phosphate buffer (pH 4.0)] was added to each well. After 20-min incubation at room temperature, 50 μL of 0.5 M ${\text{H}}_{2}{\text{SO}}_{4}$ was added to stop the reaction, and the absorbance at 450 nm of the reaction solution in each well was measured with a microplate reader.

The procedure for HRP-based ELISA of IL-6 was the same as the standard procedure for Pd@Pt ND ELISA of IL-6 except that the Pd@Pt ND-SA conjugates were substituted with HRP-SA conjugates and the substrate solution was changed to 0.8 mM TMB and 2 mM ${\text{H}}_{2}{\text{O}}_{2}$ in citrate-phosphate buffer (pH 4.0).

## 3. Results and Discussion

### 3.1. Synthesis and characterization of Pd@Pt NDs.

**Figure 1.**
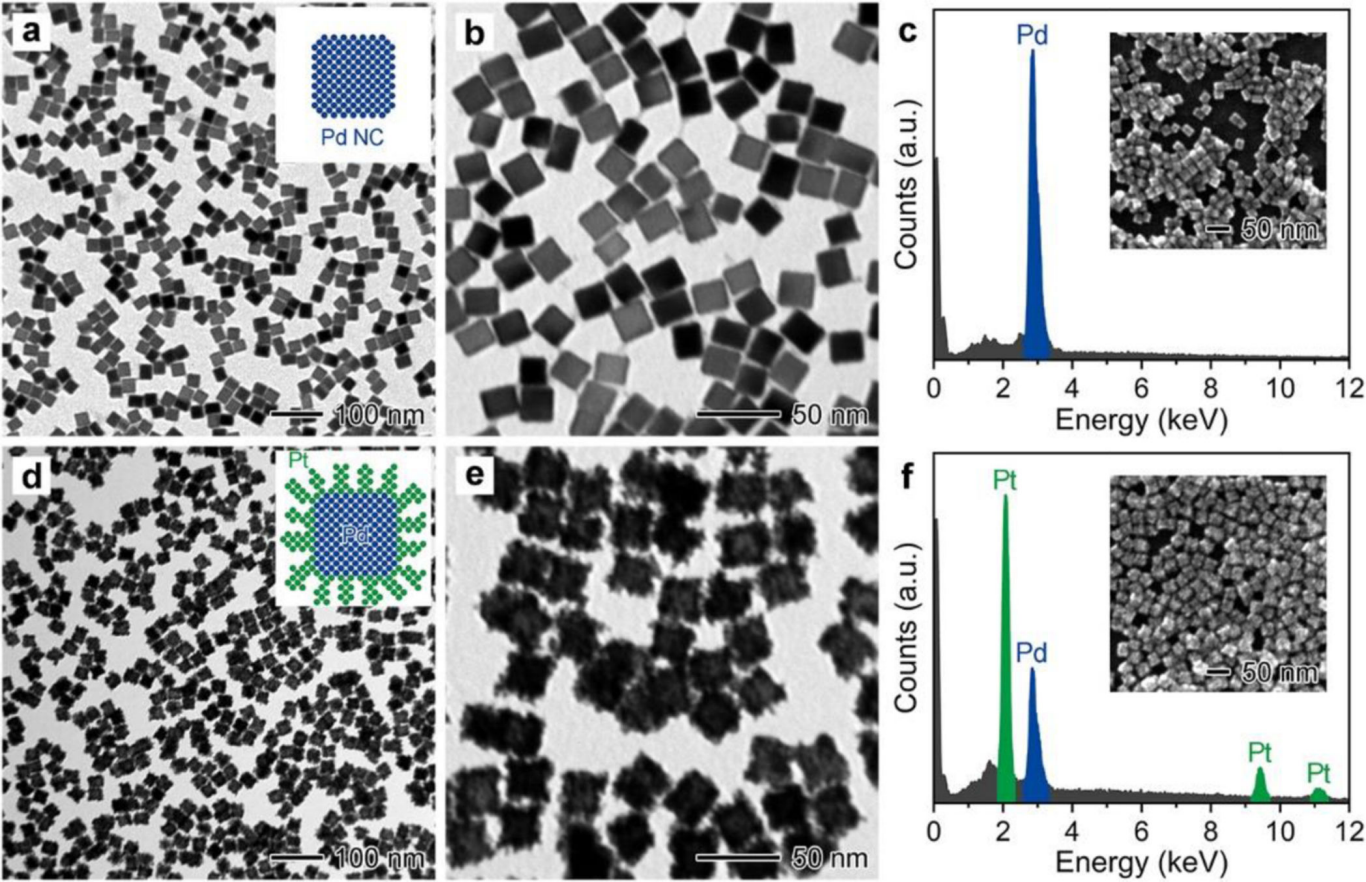
Characterization of 18 nm Pd NCs and Pd@Pt NDs. (a,b) Low- (a) and high- (b) magnification TEM images of 18 nm Pd NCs. (c) EDX spectrum of 18 nm Pd NCs deposited on a silicon substrate. (d,e) Low- (d) and high- (e) magnification TEM images of Pd@Pt NDs. (f) EDX spectrum of Pd@Pt NDs deposited on a silicon substrate. Insets in (c, f) show the corresponding scanning electron microscope (SEM) images of the region where the EDX spectra were acquired.

The Pd@Pt NDs were synthesized by rapidly depositing a mass number of Pt atoms on the surfaces of PdNCs *via* a facile seed-mediated growth method. The Pd NCs with an edge length of 18 nm were selected as the seeds because they can be easily prepared on a large scale with high uniformity, purity, and quantity through a simple one-pot synthesis (see Figure 1a-c) [50], making it conducive to producing Pd@Pt NDs of high quality. The Pt dendritic nanostructures were chosen as the shells because they can exhibit highly efficient peroxidase-like catalytic activity [43–49], allowing for producing Pd@Pt NDs with high catalytic activity. In addition, the lattice mismatch between Pd and Pt can be negligible (~0.8%) [54], and thereby Pt atoms can be more easily deposited on the surface of Pd nanocubes for the successful synthesis of Pd@Pt core@shell dendritic nanostructures. By combining the advantages of Pd NC seeds and Pt dendritic nanoshells, the resulted the Pd@Pt NDs can not only be easily large-scale prepared with high quantity, but also exhibit high peroxidase-like catalytic activity. Other sizes, shapes, and elements of noble metal nanoparticles may either require complicated synthesis procedures with low purity and uniformity or exhibit relatively low peroxidase-like catalytic activity [55–58].

In a standard synthesis of Pd@Pt NDs, three aqueous solutions including 18 nm Pd NCs as the seeds, ${\text{Na}}_{2}{\text{PtCl}}_{4}$ as the precursor, and AA as the reductant were simply mixed and heated at 80 °C for 3 h (see Materials and Methods for the detailed synthesis procedure). Figure 1d,e, show representative low- and high-magnification transmission electron microscope (TEM) images of the Pd@Pt NDs obtained from a standard synthesis, respectively. It can be observed that after the growth of Pt atoms on Pd NCs, *i)* the edge length of the Pd NCs was increased from 18 nm to 27 nm; *ii)* a layer of rough dendrite-like shells was uniformly coated on the surface of each Pd NC; and *iii)* the resulted nanoparticles presented well-preserved uniformity in size and shape as the pristine Pd NCs. The full energy-dispersive X-ray (EDX) spectra of the Pd@Pt NDs and Pd NCs confirm that the Pd@Pt NDs were primarily composed of Pd and Pt elements, while the initial 18 nm Pd NC seeds consisted of Pd element only (Figure 1c,f), indicating that the rough dendrite-like shells were mainly constructed by Pt. These results demonstrate the successful formation of Pd@Pt core@dendrite-like-shell nanostructures for the Pd@Pt NDs. The formation of dendrite-like Pt shells on Pd NC cores is mainly because Pt grows as a set of branches on Pd surfaces following the Volmer-Weber growth mode under the condition of fast reduction at relatively low temperature [59–61]. It is worth noting that the edge lengths of the Pd@Pt NDs synthesized from three different batches were measured to be 27.3 ± 1.4 nm, 27.2 ± 1.5 nm, and 26.8 ± 1.5 nm, respectively, with the coefficient of variation (CV) of 1.0% (*n* = 3, Figure S1), indicating that the Pd@Pt NDs can be readily reproduced with high uniformity. In addition, these Pd@Pt NDs can be easily scaled up 60-fold to achieve a production of 95.6 mg per batch without compromising the quality (Figure S2), while previously reported porous Pt NDs could only be produced at the scale of several milligrams per batch [43–49]. Such a large-scale production (144 mL of 6.10 nM Pd@Pt NDs) could allow 72,000 tests for the following Pd@Pt ND ELISA (100 μL of 0.122 nM per assay). Therefore, the Pd@Pt NDs designed in this work can be readily produced in large quantities with high quality, and thus they have great potential for application in the large-scale production of ELISA kits.

### 3.2. Peroxidase-like catalytic activity of Pd@Pt NDs.

**Figure 2.**
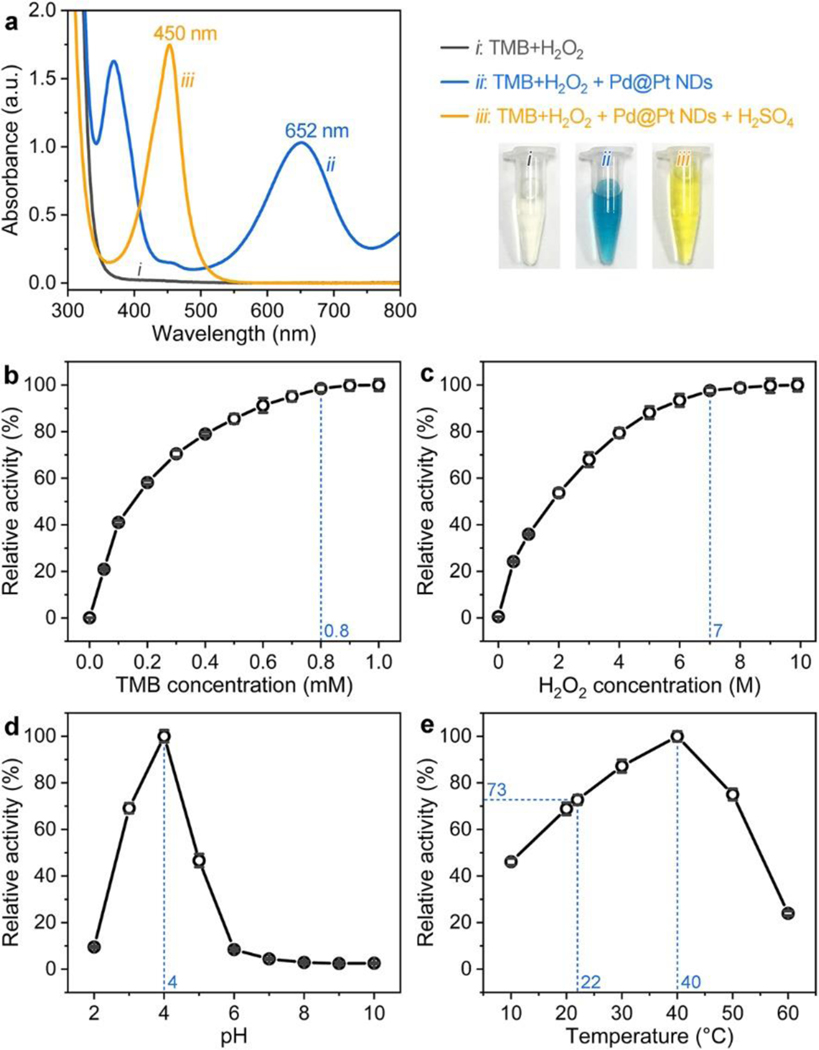
Verification and optimization of the peroxidase-like catalytic activity of Pd@Pt NDs. (a) UV-vis spectra of $\text{TMB}+{\text{H}}_{2}{\text{O}}_{2}$ substrate solution (*i*), $\left(\text{TMB}+{\text{H}}_{2}{\text{O}}_{2}\right)+\text{Pd@Pt}$ NDs solution (*ii*), and $\left(\text{TMB}+{\text{H}}_{2}{\text{O}}_{2}\right)+\text{Pd@Pt}\;{\text{ND}}_{5}+{\text{H}}_{2}{\text{SO}}_{4}$ solution (*iii*). The bottom-right panel shows the corresponding photographs taken from the three solutions. (b-e) Effect of TMB concentration (b), ${\text{H}}_{2}{\text{O}}_{2}$ concentration (c), pH (d), and temperature (e) on the peroxidase-like catalytic activity of Pd@Pt NDs. The maximum point in each curve is set as 100%.

The peroxidase-like catalytic activity of Pd@Pt NDs was then investigated through their catalysis toward the oxidation of TMB by ${\text{H}}_{2}{\text{O}}_{2}$. As shown in Figure 2a, *i)* when Pd@Pt NDs were added to $\text{TMB}+{\text{H}}_{2}{\text{O}}_{2}$ substrate solution, the reaction solution turned from colorless to blue and exhibited a new distinct absorbance peak at 652 nm; and *ii)* when ${\text{H}}_{2}{\text{SO}}_{4}$ solution was further added to stop the catalytic reaction, the color of the reaction solution changed from blue to yellow, and its absorbance peak shifted from 652 nm to 450 nm. These results indicate that the Pd@Pt NDs could catalyze the oxidation of TMB by ${\text{H}}_{2}{\text{O}}_{2}$ to produce oxidized TMB (oxTMB), which was consistent with many reported Pt-based peroxidase nanomimics, [40,43–49,51,62–64] demonstrating the intrinsic peroxidase-like catalytic property of Pd@Pt NDs. Similar to the enzyme HRP and many other peroxidase nanomimics, [30,40,43–49,51,62–64] the peroxidase-like catalytic efficiency of Pd@Pt NDs is strongly dependent on the concentrations of substrates (*e.g.*, TMB and ${\text{H}}_{2}{\text{O}}_{2}$), the pH of reaction solution, and the reaction temperature. The effects of these factors on the catalytic efficiency of Pd@Pt NDs were systematically examined, and the optimal catalytic conditions for Pd@Pt NDs toward $\text{TMB}+{\text{H}}_{2}{\text{O}}_{2}$ reaction were found to be a TMB concentration of 0.8 mM, a ${\text{H}}_{2}{\text{O}}_{2}$ concentration of 7.0 M, a reaction pH of 4.0, and a reaction temperature of 40 °C (Figure 2b-e). It should be noted that there was only a 27% reduction in the catalytic efficiency when the reaction temperature is decreased from 40 °C to room temperature (~22 °C). Considering the simplification and practicability for the use of Pd@Pt NDs in the following Pd@Pt ND ELISA, room temperature (~22 °C) was selected for the catalytic reaction throughout the experiment while the other optimal catalytic conditions remained unchanged.

**Figure 3.**
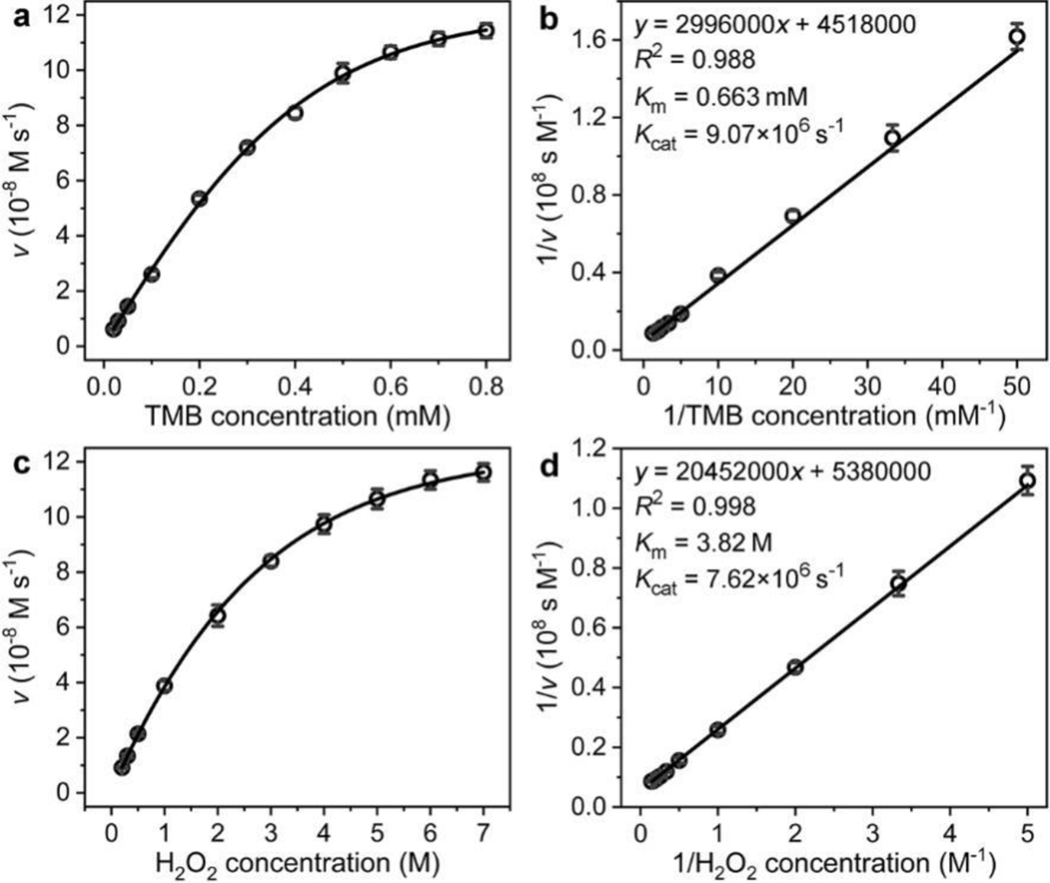
Steady-state kinetic analysis of the peroxidase-like catalytic activity of Pd@Pt NDs. (a, b) Kinetic analysis toward TMB: (a) plot of v *versus* TMB concentration, in which 7.0 M ${\text{H}}_{2}{\text{O}}_{2}$ was used; (b) double-reciprocal plot converted from (a). (c, d) Kinetic analysis toward ${\text{H}}_{2}{\text{O}}_{2}$: (c) plot of v *versus* ${\text{H}}_{2}{\text{O}}_{2}$ concentration, in which 0.8 mM TMB was used; (d) double-reciprocal plot converted from (c).

To further evaluate the peroxidase-like catalytic activity of Pd@Pt NDs, their catalytic efficiency was quantified by determining the apparent steady-state kinetic parameters for the catalytic reaction of Pd@Pt NDs toward the oxidation of TMB by ${\text{H}}_{2}{\text{O}}_{2}$. Initially, a series of *ν* for the catalytic reaction of Pd@Pt NDs toward TMB and ${\text{H}}_{2}{\text{O}}_{2}$ were measured from the steady-state kinetic assays by varying the concentration of one substrate (TMB or ${\text{H}}_{2}{\text{O}}_{2}$) while fixing the concentration of the other substrate (${\text{H}}_{2}{\text{O}}_{2}$ or TMB, see Materials and Methods for the detailed experimental procedures). Then, by plotting the v against TMB and ${\text{H}}_{2}{\text{O}}_{2}$ concentrations, typical Michaelis-Menten plots were observed for both TMB and ${\text{H}}_{2}{\text{O}}_{2}$ (Figure 3a,c). To calculate the apparent steady-state kinetic parameters of the reaction, the Michaelis-Menten plots were further converted into the double-reciprocal plots (Figure 3b,d). It can be seen that for each of the plots, the reciprocal of the *v* was in a linear relationship with the reciprocal of the substrate concentration, which could be fitted to the Lineweaver-Burk equation:

$$1/v=\left(K_{\text{m}}/V_{\max}\right)(1/[S])+1/V_{\max}$$

By coupling the linear fitting regression equation of the double-reciprocal plots with the Lineweaver-Burk equation, the $K_{m}$ and $V_{\max}$ of Pd@Pt NDs toward TMB and ${\text{H}}_{2}{\text{O}}_{2}$ could be calculated, and the $K_{\text{cat}}$ could be obtained by the equation: $K_{\text{cat}}=V_{\max}/[E]$. The calculated apparent steady-state kinetic parameters including $K_{\text{m}}$, $V_{\max}$, and $K_{\text{cat}}$ were summarized in Table 1. For comparison, the kinetic parameters for HRP [30] and many other reported peroxidase nanomimics were also listed in Table 1 and Table S1, respectively. Because the $K_{\text{cat}}$ determines the catalytic efficiency of a catalyst, which is directly correlated to the detection sensitivity of ELISA, we mainly compared the $K_{\text{cat}}$ values of Pd@Pt NDs, HRP, and other reported peroxidase nanomimics. As shown in Table 1, the $K_{\text{cat}}$ values of Pd@Pt NDs toward TMB and ${\text{H}}_{2}{\text{O}}_{2}$ were calculated to be 9.06×10^6^ s^−1^ and 7.62×10^6^ s^−1^, respectively, which were more than 2000 times larger than those of HRP, indicating that Pd@Pt NDs had much higher catalytic efficiency than HRP. More significantly, Pd@Pt NDs also showed a predominantly larger $K_{\text{cat}}$ value and thereby higher catalytic efficiency in comparison to most of the reported peroxidase nanomimics (Table S1). The higher peroxidase-like catalytic efficiency of Pd@Pt NDs could be attributed to the fact that *i)* Pt nanoparticles generally show much higher peroxidase-like catalytic activity than nanoparticles made of other elements; and *ii)* the rough dendrite-like nanosurfaces of Pd@Pt NDs provide a larger surface area per particle and therefore more active sites for the reaction. [48,65]

In addition to the high catalytic efficiency, the catalytic activities of Pd@Pt NDs remained relatively stable after they had been incubated at a range of pH values from 0 to 12 for 2 h, treated at a range of temperatures from 22 °C to 90 °C for 2 h, and stored for half a year, demonstrating the outstanding chemical, thermal, and storage stabilities of Pd@Pt NDs, respectively (Figure S3). As such, the excellent peroxidase-like catalytic property and outstanding stability of Pd@Pt NDs have geared them as ideal signal labels to substitute HRP for constructing more efficient ELISAs for ultrasensitive detection of cytokines.

### 3.3. Effect of Pt content on the peroxidase-like catalytic activity of Pd@Pt NDs.

**Figure 4.**
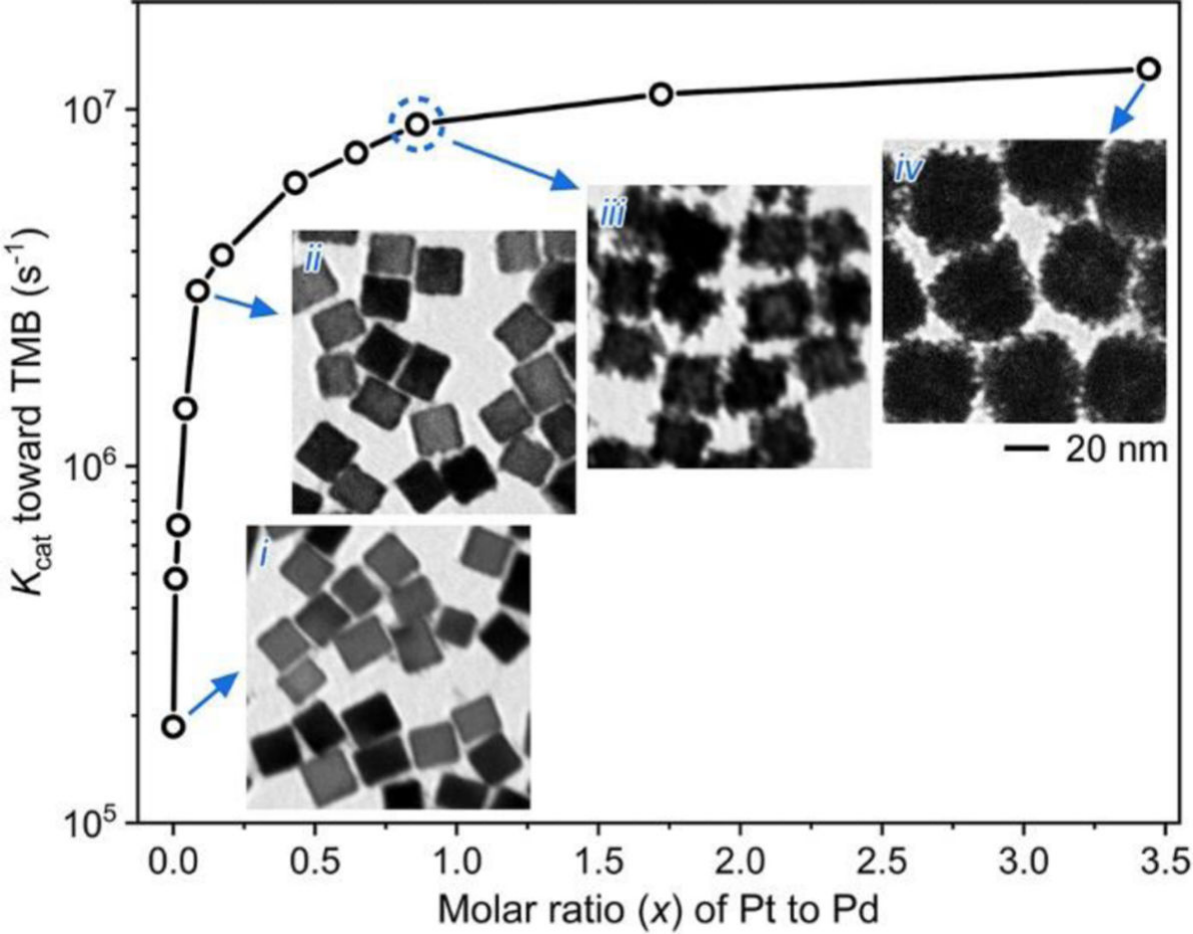
Effect of Pt content on the peroxidase-like catalytic efficiency of Pd@Pt NDs. A plot comparing the $K_{\text{cat}}$ values toward TMB of Pd@Pt NDs with different molar ratios (*x*) of Pt to Pd (*i.e.*, ${\text{Pd@Pt}}_{x}$ NDs, x=0−3.44). Insets show the TEM images of four representative ${\text{Pd@Pt}}_{x}$ NDs with x=0 (*i*), 0.0860 (*ii*), 0.860 (*iii*), and 3.44 (*iv*).

To better understand the role of Pt content in determining the peroxidase-like catalytic efficiency of Pd@Pt NDs, a set of Pd@Pt NDs with different molar ratios (*x*) of Pt to Pd (*i.e.*, ${\text{Pd@Pt}}_{x}$ NDs, *x* = 0–3.44) were prepared using the standard synthesis procedure by adjusting the amount of Pt precursor added (see Materials and Methods for the detailed synthesis procedures), and their peroxidase-like catalytic efficiencies were evaluated using the steady-state kinetic assays. Here the $K_{\text{cat}}$ value toward TMB was used to evaluate the catalytic efficiency. Figure 4 compares the $K_{\text{cat}}$ values of these ${\text{Pd@Pt}}_{x}$ NDs in the *x* range of 0–3.44. The $K_{\text{cat}}$ value increased drastically from 0 to 3.10×10^6^ in the range of *x* from 0 to 0.086. Further increase of *x* to 0.860 leaded to a steady rise of the $K_{\text{cat}}$ value to 9.06×10^6^, followed by a graduate elevation to the plateau. These results indicate that the catalytic efficiency of Pd@Pt NDs possessed a strong positive relationship with the content of Pt in Pd@Pt NDs. The insets of Figure 4 show the TEM images of four representative ${\text{Pd@Pt}}_{x}$ NDs with *x* = 0, 0.086, 0.860, and 3.44, from which it can be seen that the particle size of ${\text{Pd@Pt}}_{x}$ NDs increased with the increase of *x*. Because ${\text{Pd@Pt}}_{0.860}$ NDs (*i.e.*, the sample shown in Figure 1d,e): *i)* well balanced the catalytic efficiency of Pd@Pt NDs and the utilization efficiency of Pt element; and *ii)* had an average particle size of 27 nm with excellent dispersion stability, they were chosen as the optimized Pd@Pt NDs in this study.

### 3.4. Preparation and characterization of Pd@Pt ND-SA conjugates.

**Figure 5.**
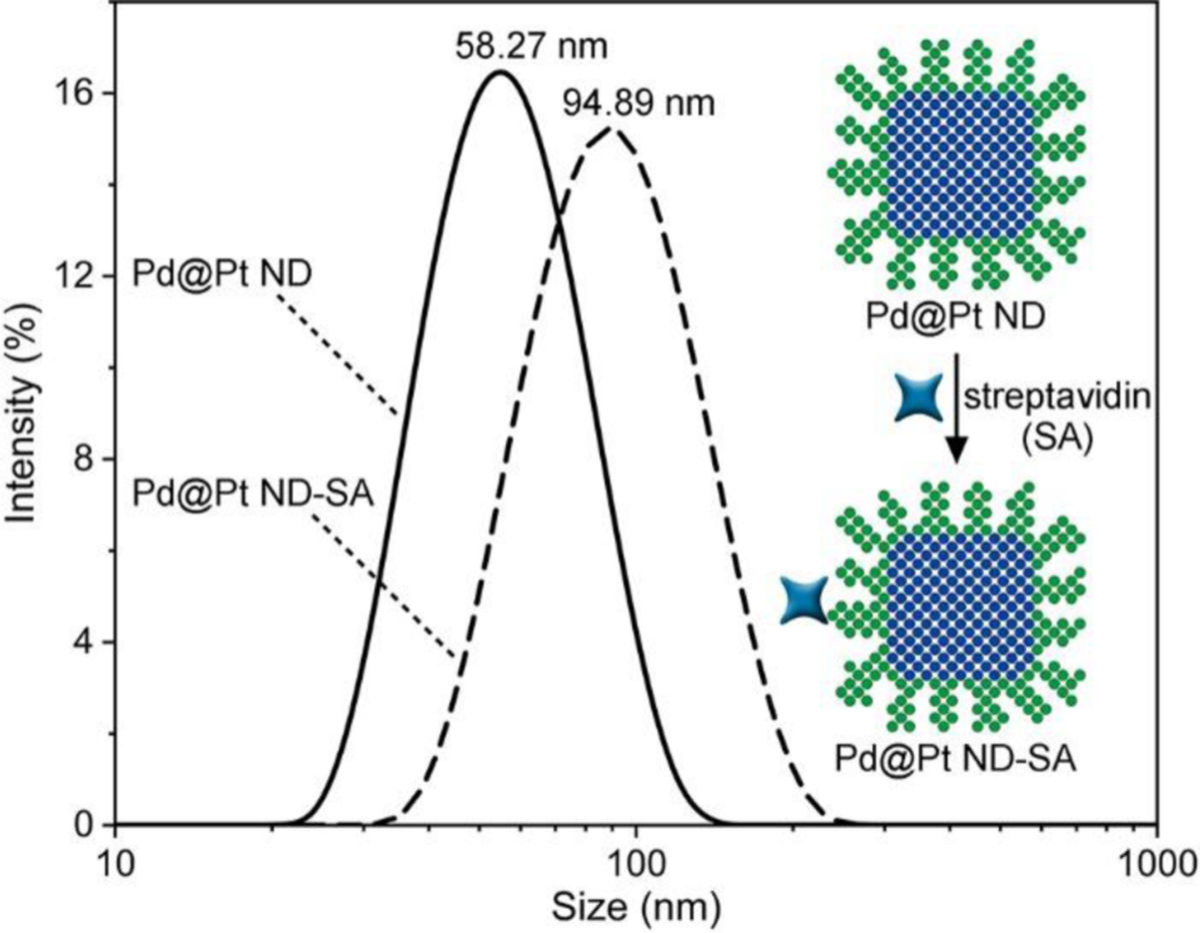
Characterization of Pd@Pt ND-SA conjugates. DLS size distributions of Pd@Pt NDs (solid curve) and Pd@Pt ND-SA conjugates (dashed curve) dispersed in DI water.

To realize the application of Pd@Pt NDs in colorimetric ELISA, Pd@Pt NDs were bio-functionalized with SA to form Pd@Pt ND-SA conjugates (see Materials and Methods for the detailed preparation procedure). To verify whether the Pd@Pt ND-SA conjugates could be successfully prepared using the above protocol, DLS analysis was carried out to measure the hydrodynamic sizes of the Pd@Pt NDs before and after the bio-functionalization of SA. As seen in Figure 5, the average hydrodynamic size of the Pd@Pt NDs increased evidently from 58.27 nm to 94.89 nm after they were conjugated with SA, suggesting the presence of SA biomolecules on the surface of the Pd@Pt NDs [66,67]. Here, the increment of the hydrodynamic size (36.62 nm) was much larger than the size of a SA biomolecule (~5 nm) [67]. The reason can be ascribed to the fact that *i)* HS-PEG-COOH polymer ($M_{\text{w}}=3,400$) is used as the linker for the conjugation of Pd@Pt NDs with streptavidin biomolecules, and thus the increase of the particle size would be the combined effect of HS-PEG-COOH polymer and SA biomolecules; and *ii)* the absorption of proteins (*e.g.*, SA) on the surface of nanoparticles (*e.g.*, Pd@Pt NDs) creates the characteristic hard and soft protein coronas, and thereby the DLS hydrodynamic size of Pd@Pt ND-SA conjugates is usually larger than their physical size [68].

To further verify the the functionalization of HS-PEG-COOH and conjugation of SA biomolecules on Pd@Pt NDs, FT-IR spectroscopy was performed on the Pd@Pt NDs before and after the functionalization and conjugation. As shown in Figure S4, no obvious peak was observed for the pristine Pd@Pt NDs in the wavenumber ranging from 750 to 4000 cm^−1^ (black curve). When HS-PEG-COOH was functionalized onto the Pd@Pt NDs, the specific peaks at 2878 cm^−1^ and 1104 cm^−1^ for HS-PEG-COOH were observed due to the C-H and C-O stretching of PEG monomer, respectively (red curve) [69,70], indicating the successful functionalization of HS-PEG-COOH on Pd@Pt NDs. When SA biomolecules were further bioconjugated to the HS-PEG-COOH-functionalized Pd@Pt NDs, the characteristic peaks at 1642 cm^−1^ and 1531 cm^−1^ related to amide I region (C-O stretching) and amide II region (N−H bending and C− stretching) of SA proteins were observed (blue curve) [70,71], revealing the successful conjugation of SA biomolecules on Pd@Pt NDs. These results demonstrate the successful preparation of Pd@Pt ND-SA conjugates using the above bio-functionalization protocol, thus providing a precondition for the following colorimetric ELISA of IL-6 based on Pd@Pt NDs as the signal labels.

### 3.5. Analytical performance of the colorimetric Pd@Pt ND ELISA for detection of IL-6.

Based on the aforementioned studies of Pd@Pt NDs including the synthesis, characterization, investigation of peroxidase-like catalytic activity, and bio-functionalization of SA, a colorimetric ELISA was developed for the detection of IL-6 using Pd@Pt NDs as the signal labels. The standard detection procedure can be found in Materials and Methods. To demonstrate the features of Pd@Pt ND ELISA, its analytical performance toward IL-6 detection was evaluated, including the sensitivity, dynamic range, reproducibility, and specificity.

**Figure 6.**
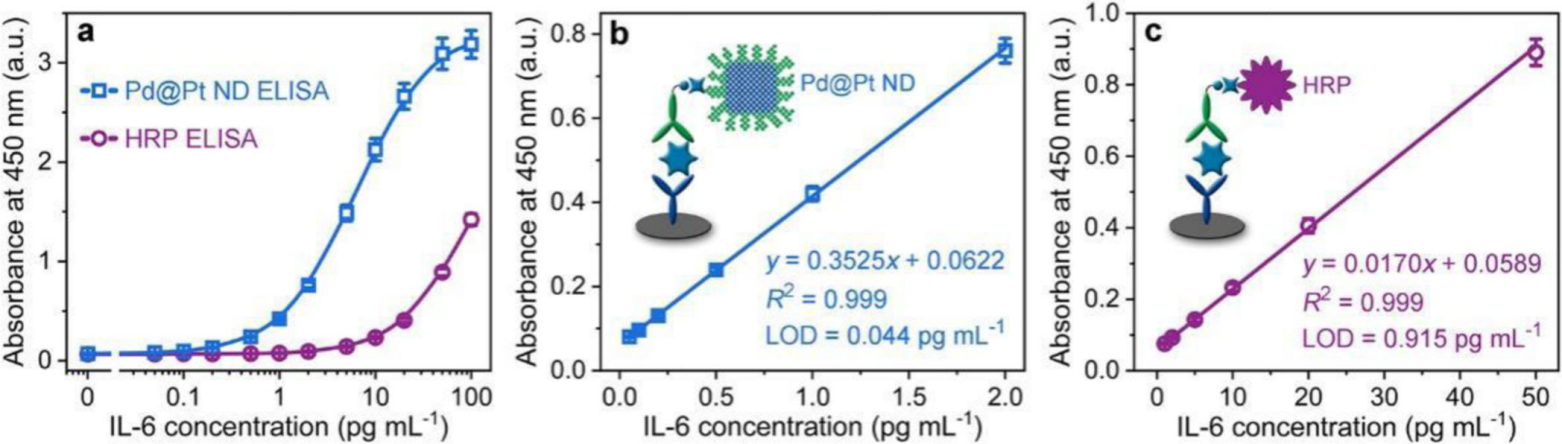
Detection of IL-6 standards using the colorimetric Pd@Pt ND ELISA (blue curve) and conventional HRP ELISA (violet curve). (a) Calibration curves of Pd@Pt ND ELISA and HRP ELISA that were generated by plotting the absorbance at 450 nm of the final detection solution against IL-6 concentration. (b,c) Linear range regions of the calibration curves for Pd@Pt ND ELISA (b) and HRP ELISA (c).

The sensitivity and dynamic range of Pd@Pt ND ELISA toward IL-6 detection were first evaluated by analyzing its response to IL-6 standards in the concentration range from 0 to 100 pg mL^-1^. The blue curve in Figure 6a shows the calibration curve of Pd@Pt ND ELISA for IL-6 detection, which was established by plotting the absorbance at 450 nm of the final detection solution against IL-6 concentration. The absorbance at 450 nm increased with the increase of IL-6 concentration ranging from 0.05 to 100 pg mL^−1^ (4 orders of magnitude), indicating a wide response range of Pd@Pt ND ELISA for IL-6 detection. As shown in Figure 6b, a high-quality linear dependence ($R^{2}=0.999$) between the absorbance and the concentration of IL-6 was obtained in the range of 0.05–2 pg mL^−1^ with an ultralow quantitative limit of detection (LOD) of 0.044 pg mL^−1^ (1.7 fM), estimated at 3SD/*k*. Here, SD is the standard deviation of the background signal (*n* = 6) and *k* is the slope of the linear regression curve. It is worth pointing out that this LOD is 21-fold lower than the LOD of conventional HRP-based ELISA, where the same antibody pair and similar procedure were employed (the violet curves in Figure 6a,c). More significantly, the achieved LOD at fM level presents the highest sensitivity among commercially available colorimetric IL-6 ELISA kits (Table S2). The high sensitivity of our colorimetric Pd@Pt ND ELISA could be ascribed to the outstanding peroxidase-like catalytic activity of Pd@Pt NDs.

It should be mentioned that the magnitude of color signal enhancement by Pd@Pt NDs relative to conventional HRP in ELISA tests was ~21 times (Figure 6), while the magnitude of color signal enhancement was ~2,000 in the case when Pd@Pt NDs and HRP were suspended in aqueous solutions (Figure 3 and Table 1). Please note that in ELISA tests, Pd@Pt NDs needed to be functionalized with HS-PEG-COOH, modified with SA, reacted with biotin on the solid-liquid interface, and finally immobilized on the solid-liquid interface (*i.e.*, the surface of ELISA microplates). Therefore, the relatively low signal enhancement in ELISA tests can be ascribed to the fact that: *i)* the functionalization of HS-PEG-COOH on Pd@Pt NDs can partly inhibit the catalytic activity of Pd@Pt NDs due to the formation of Pt-S bond [64,72]; *ii)* the modification of SA biomolecules on Pd@Pt NDs can physically hide the active Pt surface in part; *iii)* compared to HRP (4 nm in size), the larger size of Pd@Pt NDs (27 nm in edge length) impede the binding of Pd@Pt ND-SA conjugates with biotin-detection antibodies on the microplate surface because of steric hindrance effect; and *iv)* the immobilization of Pd@Pt NDs on the microplate surface also reduce the total surface area of active Pt surface as compared to the Pd@Pt NDs suspended in aqueous solution.

The reproducibility of Pd@Pt ND ELISA toward quantitative determination of IL-6 was then evaluated by repeatedly assaying three different IL-6 standards at low (0.1 pg mL^−1^), medium (1 pg mL^−1^), and high (10 pg mL^−1^) concentrations using the same- and different-batches of Pd@Pt ND-SA conjugates. The intra- and inter-batch coefficients of variation (CVs, *n* = 6) using the same- and different-batches of Pd@Pt ND-SA conjugates were determined. As summarized in Table S3, the intra-batch CVs were 5.22%, 3.82%, and 5.39% for 0.1, 1, and 10 pg mL^−1^ IL-6, respectively, while the inter-batch CVs were 8.70%, 5.06%, and 7.27% for the three concentrations of IL-6, respectively. The low CVs could be mainly due to the excellent uniformity and stability of Pd@Pt NDs, revealing the great reproducibility and repeatability of the colorimetric Pd@Pt ND ELISA and its possibility toward scale-up production for practical application.

The specificity of Pd@Pt ND ELISA toward IL-6 detection was verified by challenging the assay with other possible interfering cytokines, *e.g.*, tumor-necrosis-factor alpha (TNF-α), interferon-gamma (IFN-γ), interleukin-1 beta (IL-1β), interleukin-2 (IL-2), interleukin-8 (IL-8), and interleukin-10 (IL-10). A low concentration of target IL-6 (1 pg mL^−1^) and a high concentration of interfering cytokines (100 pg mL^−1^) were used for evaluation. As seen from Figure S5, the absorbance at 450 nm obtained from the high-concentration TNF-α, IFN-γ, IL-1β, IL-2, IL-8, and IL-10 were lower than 0.0724 a.u., which was negligible as the background signal, whereas the absorbance at 450 nm for the low-concentration IL-6 showed a significantly higher value of 0.4198 a.u., providing a highly specific detection of IL-6 by the Pd@Pt ND ELISA. It should be noted that the introduction of Pd@Pt NDs didn’t affect the specificity and could be applied to paired antibodies for any other target analytes using the conventional ELISA system.

### 3.6. Analysis of real samples and method validation.

To examine the practical application of Pd@Pt ND ELISA under clinical scenarios, we applied the assay to analyze six IL-6-spiked human serum samples. These samples were prepared by spiking IL-6 of 0.2, 0.5, 1, 2, 5, 10 pg mL^−1^ in concentration into negative human serum (*i.e.*, heat-inactivated and sterile-filtered human serum). The IL-6 level in each sample was quantified using the calibration and linear curves shown in Figure 6a,b (the blue curves). The detection results were validated with recovery analysis, which is defined as the measured level of IL-6 divided by the spiked level of IL-6 in the samples. As summarized in Table 2, the recoveries for all 6 samples were determined to be in the range of 93.65–105.53%, and the CV values for all 6 samples were smaller than 9.96% (*n* = 12). These data imply that the Pd@Pt ND ELISA maintained its high accuracy and reliability in serum with complex background components, confirming the excellent analytical performance of our colorimetric Pd@Pt ND ELISA in analyzing low-abundance IL-6 in real biological samples.

## 4. Conclusions

In conclusion, we have demonstrated an enhanced ELISA for colorimetric detection of cytokines (IL-6 as a model cytokine) with femtomolar sensitivity based on the rational design of Pd@Pt NDs with dendrite-like nanostructures as the signal labels. The Pd@Pt NDs possess outstanding peroxidase-like catalytic activity and stability, and can be scaled up for massive production with high purity, yield, and uniformity. Owing to these advantages, our colorimetric Pd@Pt ND ELISA: *i)* enables highly sensitive detection of IL-6 with ultralow LOD down to 0.044 pg mL^−1^ (1.7 fM); *ii)* exhibits excellent reproducibility and specificity; and *iii)* is suitable for large-scale production and application of the immunoassay. To the best of our knowledge, our study is the first to demonstrate a femtomolar-sensitivity ELISA for colorimetric detection of IL-6, where only one-step signal amplification is involved. The unprecedented LOD of our colorimetric Pd@Pt ND ELISA, one order of magnitude lower than the LOD of commonly used HRP-based colorimetric ELISA, allows the monitoring of lower levels of IL-6 in human serum with high accuracy and reliability. We believe that the colorimetric Pd@Pt ND ELISA presents a promising *in-vitro* diagnostic tool for quantitative detection of ultralow-level cytokines in real biological samples, and has vast applications in fundamental biomedical research and practical clinical diagnosis.

## Supplementary Material

Supporting information

## Figures and Tables

**Table 1.** Comparison of the kinetic parameters of Pd@Pt NDs and HRP toward the $\text{TMB}+{\text{H}}_{2}{\text{O}}_{2}$ reaction.^a^

Catalyst	[*E*] (M)	Substance	$K_{\text{m}}$ (M)	$V_{\max}$ (M s^−1^)	$K_{\text{cat}}$ (s^−1^)
Pd@Pt NDs	2.44×10^−14^	TMB	6.63×10^−4^	2.21×10^−7^	9.06×10^6^
	2.44×10^−14^	${\text{H}}_{2}{\text{O}}_{2}$	3.82×10^0^	1.86×10^−7^	7.62×10^6^
HRP (ref. 30)	2.5×10^−11^	TMB	4.3×10^−4^	1.0×10^−7^	4.0×10^3^
	2.5×10^−11^	${\text{H}}_{2}{\text{O}}_{2}$	3.7×10^−3^	8.7×10^−8^	3.5×10^3^

a [*E*] is the catalyst concentration, $K_{\text{m}}$ is the Michaelis-Menten constant, $V_{\max}$ is the maximal reaction rate, and $K_{\text{cat}}$ is the catalytic constant, where $K_{\text{cat}}=V_{\max}/[E]$.

**Table 2.** Analytical recoveries of Pd@Pt ND ELISA in detecting IL-6-spiked human serum samples.

Sample no.	IL-6 level spiked (pg mL^−1^)	IL-6 level meaured (pg mL^−1^)	CV (%, *n* = 12)	Recovery (%)
1	0.2	0.19	9.96	94.69
2	0.5	0.47	7.55	93.65
3	1	0.98	5.60	97.55
4	2	1.92	6.70	96.12
5	5	5.28	8.86	105.53
6	10	9.97	8.34	99.74

## Data Availability

Data is contained within the article.
