# Supplementary material for: Pd@Pt Nanodendrites as Peroxidase Nanomimics for Enhanced Colorimetric ELISA of Cytokines with Femtomolar Sensitivity
Source: Chemosensors (Basel). Author manuscript; Available in PMC 2023 Nov 30. (PMC10688776; doi:10.3390/chemosensors10090359)
Supplement: Supporting information [file NIHMS1900320-supplement-Supporting_information.docx]

Supporting Information for

**Pd@Pt Nanodendrites as Peroxidase Nanomimics for Enhanced Colorimetric ELISA of Cytokines with Femtomolar Sensitivity**

Zhuangqiang Gao,^*^ Chuanyu Wang, Jiacheng He, and Pengyu Chen^*^

*Materials Research and Education Center, Materials Engineering, Department of Mechanical Engineering, Auburn University, Auburn, Alabama 36849, United States*

^*^*Corresponding author. E-mail: [zhuangqiang.gao@hotmail.com](mailto:zhuangqiang.gao@hotmail.com) and [pengyuc@auburn.edu](mailto:pengyuc@auburn.edu)*

**Additional Materials and Methods**

*S1. Chemicals and Materials.*

Sodium tetrachloropalladate(II) (Na_2_PdCl_4_, 98%), potassium bromide (KBr, ≥99%), L-ascorbic acid (AA, ≥99%), poly(vinylpyrrolidone) (PVP, *M*_W_≈55,000), sodium hexachloroplatinate(IV) hexahydrate (Na2PtCl6·6H2O, 98%), 3,3',5,5'-tetramethylbenzidine (TMB, >99%), and horseradish peroxidase (HRP) were obtained from Millipore Sigma. Hydrogen peroxide solution (30 wt% in H2O), sodium bicarbonate (NaHCO_3_, ≥99.7%), sodium carbonate (Na_2_CO_3_, ≥99.5%), sodium phosphate dibasic (Na_2_HPO_4_, ≥99.0%), sodium chloride (NaCl, ≥99.0%), potassium phosphate monobasic (KH_2_PO_4_, ≥99.0%), potassium chloride (KCl, ≥99.0%), Tween 20, citric acid, bovine serum albumin (BSA, ≥98%), sucrose, and streptavidin (SA) were all obtained from VWR. Thiol-PEG-Carboxyl (*M*_w_ ≈ 3400, HS-PEG-COOH) was obtained from Laysan Bio, Inc. N-ethyl-N′-(3- (dimethylamino)propyl)carbodiimide hydrochloride (EDC, ≥98.0%) and N-hydroxysulfosuccinimide sodium salt (NHS, ≥98.0%) were obtained from Tokyo Chemical Industry Co., Itd. Human interleukin-6 (IL-6), mouse anti-cytokine capture antibody (CAb), biotin-conjugated detection antibodies (Biotin-DAb), and HRP-conjugated SA (HRP-SA) were obtained from Fisher Scientific, Inc. Deionized water (DI) with a resistivity of 18.2 MΩ·cm was used throughout the experiments. 10 mM Carbonate-bicarbonate buffer (pH 9.6) was prepared by dissolving 1.59 g of Na_2_CO_3_ and 2.93 g of NaHCO_3_ in 1.0 L of DI water. 10 mM phosphate-buffered saline (PBS, pH 7.4) was prepared by dissolving 1.15 g of Na_2_HPO_4_, 0.24 g of KH_2_PO_4_, 0.2 g of KCl, and 8.0 g of NaCl in 1.0 L of DI water. Citrate-phosphate buffer (pH 4.0) was prepared by 11.825 g of Na_2_HPO_4_ and 2.3650 g citric acid in 1.0 L of DI water. Washing buffer was PBS (pH 7.4) containing 0.05% Tween 20 (PBST). Block-fix buffer was PBST containing 1% BSA and 15% sucrose. Dilution buffer was PBST containing 1% BSA.

*S2. Characterizations.*

The transmission electron microscope (TEM) images were taken using a Zeiss EM10 transmission electron microscope. The scanning electron microscopy (SEM) images and EDX spectra were obtained using a JEOL JSM-7000F microscope. The UV-vis spectra and kinetic curves of apparent steady-state kinetic assays were recorded using an Ultrospec 2100 pro UV-vis spectrophotometer (Amersham Biosciences). Dynamic light scattering (DLS) data were obtained using a Zetasizer Nano ZS90, Malvern. The absorbance of samples in wells of microplates was measured using a SpectraMax iD3 Multi-Mode Microplate Reader (Molecular Devices). Fourier-transform infrared (FT-IR) spectra were performed using a PerkinElmer Spectrum 400 FT-IR/FT-NIR Spectrometer.


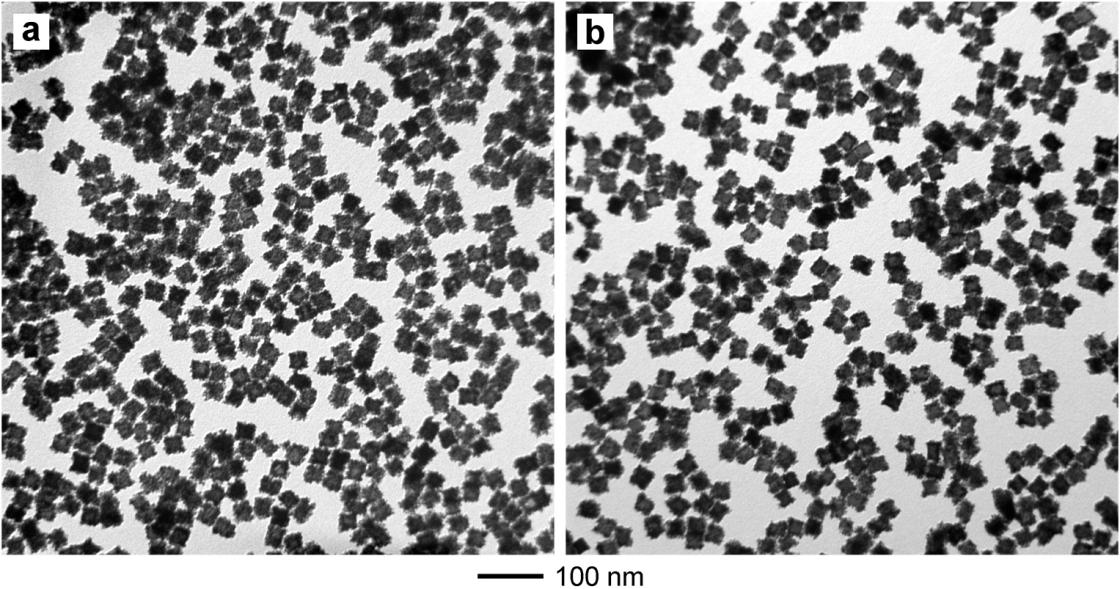


**Figure S1.** TEM images of Pd@Pt NDs synthesized from two other batches in addition to the batch shown in Figure 1d,e. It can be seen that these two-batch Pd@Pt NDs presented the same dendrite-like morphology as the Pd@Pt NDs shown in Figure 1d,e. The edge lengths of these two-batch Pd@Pt NDs and the Pd@Pt NDs shown in Figure 1d,e were measured to be 27.3 ± 1.4 nm, 27.2 ± 1.5 nm, and 26.8 ± 1.5 nm, respectively, with the coefficient of variation (CV) of 1.0% (*n* = 3). These results indicate that the Pd@Pt NDs can be readily reproduced with high uniformity.


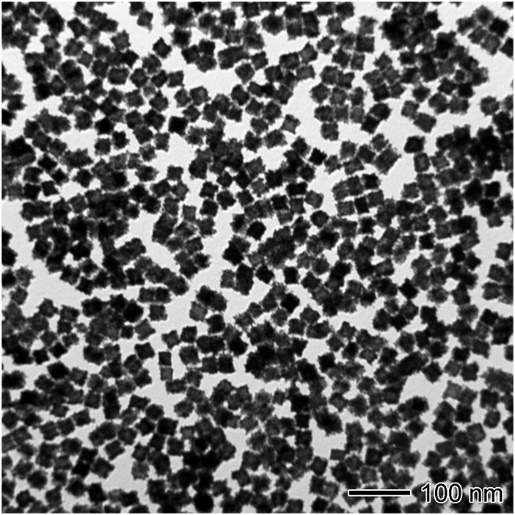


**Figure S2.** TEM image of Pd@Pt NDs prepared from 60-fold scale-up synthesis. Please see Materials and Methods for the detailed synthesis procedures.


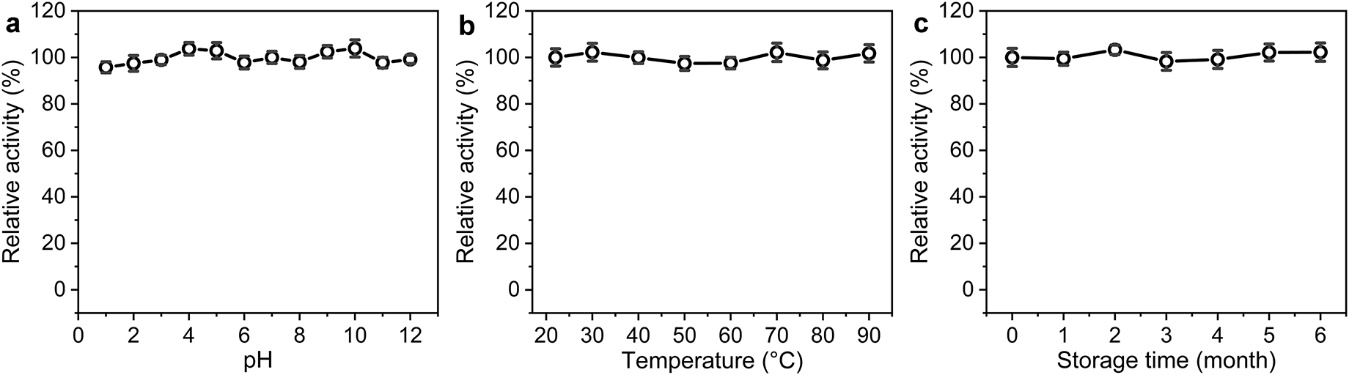


**Figure S3.** Stability evaluation for the peroxidase-like catalytic activity of Pd@Pt NDs. Relative catalytic activities of Pd@Pt NDs after incubation with acid or base (pH 1-12) for 2 h (a), treatment with heat (22-90 °C) for 2 h (b), and storage for different time (0-6 months, c), in which the activity at pH 4.0, 22 ℃, and 0 month was set as 100%.


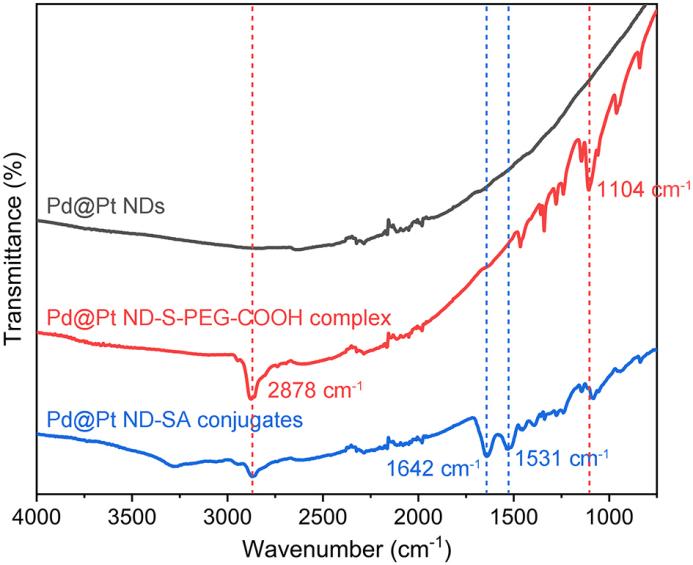


**Figure S4.** FT-IR spectra of Pd@Pt NDs (black curve), Pd@Pt ND-S-PEG-COOH complex (red curve), and Pd@Pt ND-SA conjugates (blue curve).


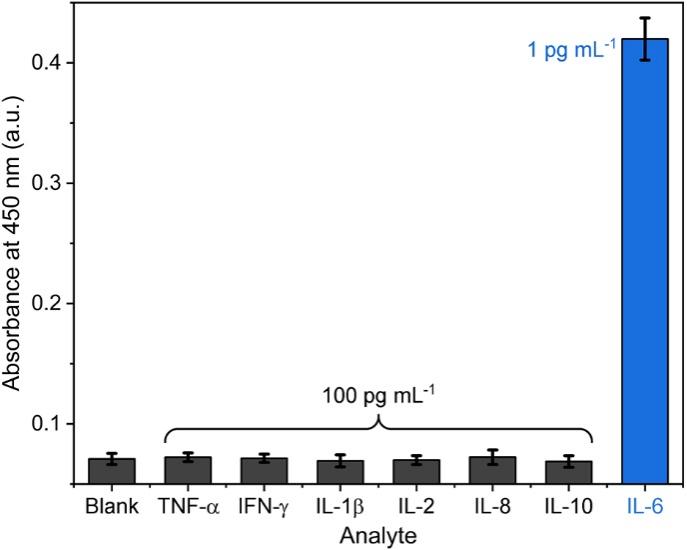


**Figure S5**. Specificity of Pd@Pt ND ELISA toward IL-6 detection. Bar graph showing the intensity of the detection signal for detection of IL-6 (1 pg mL^-1^) and interfering cytokines including TNF-α, IFN-γ, IL-1β, IL-2, IL-8, and IL-10 (100 pg mL^-1^) using Pd@Pt ND ELISA.

**Table S1.** Comparison of the kinetic parameters of various catalysts toward the TMB+H_2_O_2_ reaction.*^a^*

| Catalyst | Size  (nm) | [*E*]  (M) | Substance | *K*_m_  (M) | *V*_max_  (M s^-1^) | *K*_cat_  (s^-1^) | Refs. |
| --- | --- | --- | --- | --- | --- | --- | --- |
| HRP | N/A | 2.5×10^-11^ | TMB | 4.3×10^-4^ | 1.0×10^-7^ | 4.0×10^3^ | [S1] |
|  |  | 2.5×10^-11^ | H_2_O_2_ | 3.7×10^-3^ | 8.7×10^-8^ | 3.5×10^3^ |  |
| Fe_3_O_4_  particles | 300  (diameter) | 1.1×10^-12^ | TMB | 9.8×10^-5^ | 3.4×10^-8^ | 3.0×10^4^ | [S1] |
|  |  | 1.1×10^-12^ | H_2_O_2_ | 1.5×10^-1^ | 9.8×10^-8^ | 8.6×10^4^ |  |
| Co_3_O_4_  cubes | 20  (edge length) | 3.4×10^-10^ | TMB | 3.7×10^-5^ | 6.3×10^-8^ | 1.8×10^2^ | [S2] |
|  |  | 3.4×10^-10^ | H_2_O_2_ | 1.4×10^-1^ | 1.2×10^-7^ | 3.5×10^2^ |  |
| MnO_2_  particles | 4.5  (diameter) | 3.0×10^-8^ | OPD | 3.1×10^-4^ | 8.2×10^-8^ | 2.7×10^0^ | [S3] |
|  |  | 3.0×10^-8^ | H_2_O_2_ | 1.2×10^-4^ | 5.7×10^-8^ | 1.9×10^0^ |  |
| V_2_O_5_  wires | 100×500  (width×length) | 1.1×10^-4^ | ABTS | 4.0×10^-7^ | 2.8×10^-1^ | 2.5×10^3^ | [S4] |
|  |  | N/A | H_2_O_2_ | 2.9×10^-6^ | N/A | N/A |  |
| Au  particles | 40  (diameter) | 6.7×10^-12^ | TMB | N/A | 4.8×10^-8^ | 7.2×10^3^ | [S5] |
|  |  | N/A | H_2_O_2_ | N/A | N/A | N/A |  |
| Ru  frames | 10  (diameter) | 1.1×10^-12^ | TMB | 6.0×10^-5^ | 1.3×10^-7^ | 1.3×10^4^ | [S6] |
|  |  | 1.1×10^-12^ | H_2_O_2_ | 3.8×10^-1^ | 7.4×10^-8^ | 7.0×10^3^ |  |
| Au@Pt  rods | 30×70  (width×length) | 1.3×10^-11^ | TMB | 2.7×10^-5^ | 1.8×10^-7^ | 1.4×10^4^ | [S7] |
|  |  | N/A | H_2_O_2_ | N/A | N/A | N/A |  |
| Pt  particles | 5-7  (diameter) | 8.1×10^-11^ | TMB | 1.2×10^-4^ | 1.3×10^-6^ | 2.3×10^4^ | [S8] |
|  |  | 8.1×10^-11^ | H_2_O_2_ | 7.7×10^-1^ | 1.9×10^-6^ | 1.6×10^4^ |  |
| Pd  cubes | 18  (edge length) | 1.4×10^-12^ | TMB | 5.4×10^-5^ | 9.7×10^-8^ | 6.9×10^4^ | [S9] |
|  |  | 1.4×10^-12^ | H_2_O_2_ | 7.0×10^-1^ | 6.5×10^-8^ | 4.6×10^4^ |  |
| Pd-Ru cubes | 20  (edge length) | N/A | TMB | N/A | N/A | 4.8×10^5^ | [S10] |
|  |  | N/A | H_2_O_2_ | N/A | N/A | N/A |  |
| Pt  cubes | 7.4  (edge length) | 4.1×10^-13^ | TMB | 7.3×10^-4^ | 3.3×10^-7^ | 8.2×10^5^ | [S11] |
|  |  | N/A | H_2_O_2_ | N/A | N/A | N/A |  |
| Au@Pt  particles | 42  (diameter) | 6.3×10^-14^ | TMB | N/A | 9.7×10^-8^ | 1.5×10^6^ | [S5] |
|  |  | N/A | H_2_O_2_ | N/A | N/A | N/A |  |
| Pd-Ir  cubes | 19.2  (edge length) | 3.4×10^-14^ | TMB | 1.3×10^-4^ | 6.5×10^-8^ | 1.9×10^6^ | [S9] |
|  |  | 3.4×10^-14^ | H_2_O_2_ | 3.4×10^-1^ | 5.1×10^-8^ | 1.5×10^6^ |  |
| Pd@Pt  cubes | 20  (edge length) | 2.6×10^-13^ | TMB | 3.4×10^-4^ | 6.0×10^-7^ | 2.3×10^6^ | [S12] |
|  |  | 2.6×10^-13^ | H_2_O_2_ | 7.7×10^-1^ | 5.8×10^-7^ | 2.2×10^6^ |  |
| Concave Pt cubes | 44  (diameter) | 2.5×10^-14^ | TMB | N/A | 1.5×10^-7^ | 6.0×10^6^ | [S13] |
|  |  | 2.5×10^-14^ | H_2_O_2_ | N/A | 1.3×10^-7^ | 5.1×10^6^ |  |
| Pd@Pt NDs | 27  (edge length) | 2.44×10^-14^ | TMB | 6.63×10^-4^ | 2.21×10^-7^ | 9.06×10^6^ | This work |
|  |  | 2.44×10^-14^ | H_2_O_2_ | 3.82×10^0^ | 1.86×10^-7^ | 7.62×10^6^ |  |

*^a^*[*E*] is the catalyst concentration, *K*_m_ is the Michaelis-Menten constant, *V*_max_ is the maximal reaction rate, *K*_cat_ is the catalytic constant, where *K*_cat_=*V*_max_/[*E*], and N/A is “not applicable”. The mechanism behind the catalysis of Pd@Pt NDs toward the TMB+H_2_O_2_ reaction was proposed as shown in the following schematic, in which the catalytic effect of Pd@Pt NDs on H_2_O_2_ decomposition could be better understood [S8,S9].


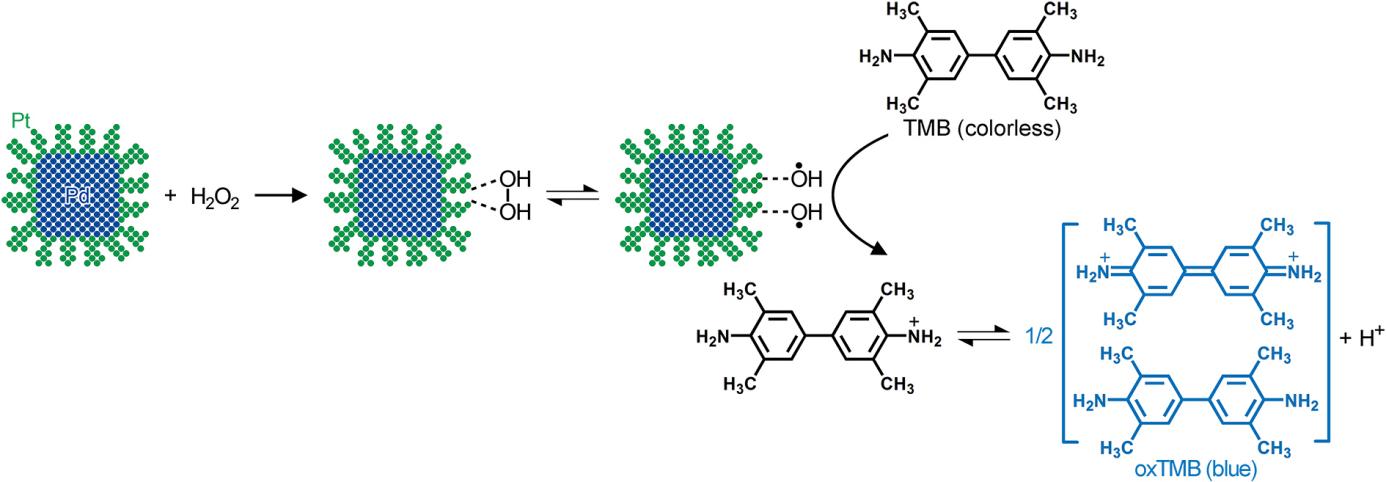


**Table S2.** Comparison of the limits of detection (LODs) of colorimetric Pd@Pt ND ELISA with some different commercial colorimetric ELISA kits in IL-6 detection.

| Detection method | Manufacturer | Catalog number | LOD  (pg mL^-1^) | LOD  (fM) |
| --- | --- | --- | --- | --- |
| Commercial ELISA kits | Fisher Scientific | KAC1261 | 2 | 76.9 |
|  |  | KHC0061 | 2 | 76.9 |
|  |  | EH2IL6 | 1 | 38.5 |
|  |  | BMS213-2 | 0.92 | 35.4 |
|  | Milllipore Sigma | RAB0306-1KT | 3 | 115.4 |
|  |  | RAB0307-1KT | 3 | 115.4 |
|  | Abcam | ab100573 | 30 | 1,153.8 |
|  |  | ab100572 | 3 | 115.4 |
|  |  | ab46027 | 2 | 76.9 |
|  |  | ab178013 | 1.6 | 61.5 |
|  |  | ab46042 | 0.8 | 30.8 |
|  | R&D Systems | QK206 | 2.95 | 113.5 |
|  |  | D6050 | 0.7 | 26.9 |
| Pd@Pt ND ELISA | This work | This work | 0.044 | 1.7 |

**Table S3.** Intra- and inter-batch coefficients of variation (CVs, *n* = 6) of Pd@Pt ND ELISA in detecting 0.1, 1, and 10 pg mL^-1^ IL-6 standards.

|  | IL-6 conc. (pg mL^-1^) | Absorbance at 450 nm (a.u.) | | | | | | Mean  (a.u.) | Standard deviation (SD, a.u.) | CV (%, n = 6) |
| --- | --- | --- | --- | --- | --- | --- | --- | --- | --- | --- |
|  |  | 1 | 2 | 3 | 4 | 5 | 6 |  |  |  |
| Intra-batch assay | 0.1 | 0.0992 | 0.0981 | 0.0911 | 0.1020 | 0.0992 | 0.0894 | 0.0965 | 0.0050 | 5.22 |
|  | 1 | 0.4276 | 0.4005 | 0.4325 | 0.4105 | 0.4390 | 0.4044 | 0.4191 | 0.0160 | 3.82 |
|  | 10 | 2.1794 | 1.9950 | 2.2002 | 2.2437 | 2.1337 | 1.9646 | 2.1194 | 0.1142 | 5.39 |
| Inter-batch assay | 0.1 | 0.1052 | 0.1024 | 0.0990 | 0.0891 | 0.0833 | 0.0943 | 0.0956 | 0.0083 | 8.70 |
|  | 1 | 0.4447 | 0.4303 | 0.4253 | 0.4052 | 0.3862 | 0.4328 | 0.4208 | 0.0213 | 5.06 |
|  | 10 | 2.2067 | 2.3171 | 2.0749 | 2.0527 | 1.9167 | 2.2948 | 2.1438 | 0.1558 | 7.27 |

**REFERENCES**

S1. Gao, L.; Zhuang, J.; Nie, L.; Zhang, J.; Zhang, Y.; Gu, N.; Wang, T.; Feng, J.; Yang, D.; Perrett, S.; Yan, X. Intrinsic peroxidase-like activity of ferromagnetic nanoparticles. *Nat. Nanotechnol.* **2007,** *2*, 577-583.

S2. Mu, J.; Wang, Y.; Zhao, M.; Zhang, L. Intrinsic peroxidase-like activity and catalase-like activity of Co_3_O_4_ nanoparticles. *Chem. Comm.* **2012,** *48*, 2540-2542.

S3. Liu, X.; Wang, Q.; Zhao, H.; Zhang, L.; Su, Y.; Lv, Y. Bsa-templated MnO_2_ nanoparticles as both peroxidase and oxidase mimics. *Analyst* **2012,** *137*, 4552-4558.

S4. André, R.; Natálio, F.; Humanes, M.; Leppin, J.; Heinze, K.; Wever, R.; Schröder, H.-C.; Müller, W.E.G.; Tremel, W. V_2_O_5_ nanowires with an intrinsic peroxidase-like activity. *Adv. Funct. Mater.* **2011,** *21*, 501-509.

S5. Gao, Z.; Ye, H.; Tang, D.; Tao, J.; Habibi, S.; Minerick, A.; Tang, D.; Xia, X. Platinum-decorated gold nanoparticles with dual functionalities for ultrasensitive colorimetric in vitro diagnostics. *Nano Lett.* **2017,** *17*, 5572-5579.

S6. Ye, H.; Mohar, J.; Wang, Q.; Catalano, M.; Kim, M. J.; Xia, X. Peroxidase-like properties of ruthenium nanoframes. *Sci. Bull.* **2016,** *61*, 1739-1745.

S7. He, W.; Liu, Y.; Yuan, J.; Yin, J.-J.; Wu, X.; Hu, X.; Zhang, K.; Liu, J.; Chen, C.; Ji, Y.; Guo, Y. Au@Pt nanostructures as oxidase and peroxidase mimetics for use in immunoassays. *Biomaterials* **2011,** *32*, 1139-1147.

S8. Gao, Z.; Xu, M.; Hou, L.; Chen, G.; Tang, D. Irregular-shaped platinum nanoparticles as peroxidase mimics for highly efficient colorimetric immunoassay. *Anal. Chim. Acta* **2013,** *776*, 79-86.

S9. Xia, X.; Zhang, J.; Lu, N.; Kim, M. J.; Ghale, K.; Xu, Y.; McKenzie, E.; Liu, J.; Ye, H. Pd–Ir core–shell nanocubes: A type of highly efficient and versatile peroxidase mimic. *ACS Nano* **2015,** *9*, 9994-10004.

S10. Wan, S.; Wang, Q.; Ye, H.; Kim, M. J.; Xia, X. Pd–Ru bimetallic nanocrystals with a porous structure and their enhanced catalytic properties. *Part. Part. Syst. Charact.* **2018,** *35*, 1700386.

S11. Ye, H.; Liu, Y.; Chhabra, A.; Lilla, E.; Xia, X. Polyvinylpyrrolidone (PVP)-capped Pt nanocubes with superior peroxidase-like activity. *ChemNanoMat* **2017,** *3*, 33-38.

S12. Davidson, E.; Xi, Z.; Gao, Z.; Xia, X. Ultrafast and sensitive colorimetric detection of ascorbic acid with Pd-Pt core-shell nanostructure as peroxidase mimic. *Sens. Int.* **2020,** *1*, 100031.

S13. Gao, Z.; Lv, S.; Xu, M.; Tang, D. High-index {hk0} faceted platinum concave nanocubes with enhanced peroxidase-like activity for an ultrasensitive colorimetric immunoassay of the human prostate-specific antigen. *Analyst* **2017,** *142*, 911-917.
